# The 3-Biomarker Classifier—A Novel and Simple Molecular Risk Score Predicting Overall Survival in Patients with Colorectal Cancer

**DOI:** 10.3390/cancers16183223

**Published:** 2024-09-22

**Authors:** Nathaniel Melling, Mohammad H. Fard-Aghaie, Claudia Hube-Magg, Martina Kluth, Ronald Simon, Michael Tachezy, Tarik Ghadban, Matthias Reeh, Jakob R. Izbicki, Guido Sauter, Katharina Grupp

**Affiliations:** 1 Department of General, Visceral and Thoracic Surgery, University Medical Center Hamburg-Eppendorf, Martinistr. 52, 20246 Hamburg, Germany; n.melling@uke.de (N.M.); m.tachezy@uke.de (M.T.); t.ghadban@uke.de (T.G.); m.reeh@uke.de (M.R.); izbicki@uke.de (J.R.I.); k.grupp@uke.de (K.G.); 2 Institute of Pathology, University Medical Center Hamburg-Eppendorf, Martinistr. 52, 20246 Hamburg, Germany; c.hube@uke.de (C.H.-M.); m.kluth@uke.de (M.K.); r.simon@uke.de (R.S.); g.sauter@uke.de (G.S.)

**Keywords:** colorectal carcinoma, H2BUB1, RBM3, Ki-67

## Abstract

**Simple Summary:**

Colorectal cancer is one of the leading causes of cancer-related deaths worldwide. Traditional methods for predicting patient outcomes rely heavily on the physical characteristics of tumors. Our research aims to develop a new, simple risk score that uses three specific molecular markers found in tumor tissues. By examining the presence and levels of these markers, we hope to better predict which patients have a higher risk of poor outcomes. This can help doctors make more informed decisions about treatment options. Our findings may lead to improved survival rates by identifying high-risk patients who need more aggressive treatment and sparing low-risk patients from unnecessary procedures.

**Abstract:**

Introduction: Several new molecular markers in colorectal carcinomas have been discovered; however, classical histopathological predictors are still being used to predict survival in patients. We present a novel risk score, which uses molecular markers, to predict outcomes in patients with colorectal carcinoma. Methods: The immunohistochemistry of tissue micro arrays was used to detect and quantify H2BUB1, RBM3 and Ki-67. Different intensities of staining were categorized for these markers and a score was established. A multivariate analysis was performed and survival curves were established. Results: 1791 patients were evaluated, and multivariate analysis revealed that our risk score, the 3-biomarker classifier, is an independent marker to predict survival. We found a high risk-score to be associated with dismal median survival for the patients. Conclusions: A more personalized score might be able to better discriminate low- and high-risk patients and suggest adjuvant treatment compared to classical pathological staging. Our score can serve as a tool to predict outcomes in patients suffering from colorectal carcinoma.

## 1. Introduction

Colorectal carcinoma (CRC) is one of the leading causes of cancer-related death in Western countries. In 2020, colorectal carcinomas ranked second regarding mortality, with 935,000 estimated deaths worldwide [1].

Classical histopathological staging (TNM) dictates therapy algorithms and is used to predict outcomes in patients. A recommendation of adjuvant therapy is usually based on this staging, with the nodal status being the most significant criteria for postoperative therapy [2]. In hepatic metastasized colorectal carcinoma, the mutation of RAS and BRAF are associated with a poorer prognosis for the patient [3]. In R0-resected CRCs, several predictive molecular parameters were proposed; however, routine measurements were not recommended. Only in UICC Stage II-CRCs is discrimination between microsatellite stability and instability used to recommend adjuvant therapy (as proposed in the German S3-Guideline Colorectal Carcinoma) [4]. In patients with nodal negative CRCs, prognosis is usually favorable; however, the metastatic potential of the disease remains unclear, as approximately 30% of patients with UICC Stage I and II will develop metastases [5]. Further insights into molecular markers in CRCs might improve decision making and answer the question of which patients will benefit from adjuvant therapy.

Our study group has been examining this problem intensively in the last few years and has already found various markers that appear to have prognostic, and maybe even therapeutic, relevance. Examples for routinely available molecular markers, which we were able to link to patient outcomes [6,7,8], are histone H2B monoubiquitination (H2BUB1), RNA-binding motif protein 3 (RBM3) and Ki-67. H2BUB1 does not only control mRNA processing and act as a transcription regulator, but it also plays a pivotal role in tumor suppression [9,10]. RBM3 is believed to serve as a proto-oncogene and several studies have reported unfavorable outcomes in different cancer types when expression was decreased [11,12,13,14].

The antigen Ki-67 is universally used to quantify the replication in tumor cells via immunohistochemistry [15]. Currently, Ki-67 is only used in gastrointestinal neuroendocrine tumors, and is established as a predictive and therapeutical marker; however, it has not yet played a role in the management of CRCs, but our data suggest a predictive value for Ki-67 [7,16].

Scores are able to predict the outcome of and influence patient care. For clinical practice, these scores must be simple and also easily accessible. To our knowledge, scores including the above-mentioned markers are not yet established.

We present a novel and simple risk score that utilizes the routinely available molecular markers H2BUB1, RBM3 and Ki-67 (3-biomarker classifier). The 3-biomarker classifier is able to predict and stratify outcomes in patients with colorectal carcinoma.

## 2. Methods

### 2.1. Patients and Data Retrieval

All samples were collected after surgical resection and the samples were collected immediately post-surgery, prior to any adjuvant treatment. Tissue microarray construction and immunohistochemistry regarding H2BUB1, RBM3 and Ki-67 were previously published. We refer to this data regarding the material and methods [6,7,8]. In brief, two different tissue microarrays with a total of 1800 CRC samples were included in these studies. The first tissue microarray was manufactured from resection specimens of 1420 CRC patients at the Institute of Pathology of the University Hospital of Basel. Raw survival data were obtained from the responsible physicians for all of the 1420 patients. The median follow-up time was 46 months (range of 1–152 months).

The second tissue microarrays included samples from 380 CRC patients, whose tumor resection specimens were examined at the Institute of Pathology of the University Medical Center, Hamburg-Eppendorf. For these tissue microarrays as well, raw survival data were available for all of the 380 patients, with a median follow-up period of 36 months (range of 1–179 months). Patient information and clinical data, such as age, sex, localization and the type of tumor, the pTNM-stage and the carcinoma grade were retrospectively retrieved from clinical and pathological databases (Table 1). Follow-up data were obtained from local cancer register boards or via attending physicians. For statistical analyses, tumor localizations were grouped as follows: right-sided cancer (cecum, ascending colon), cancer of the transverse colon, cancer of the left-sided colon (descending and sigmoid colon), rectum.

For statistical analyses, the H2BUB1 and RBM3 staining results were categorized into three groups [6,8]: tumors without any staining were considered “negative”. Tumors with 1+ or 2+ staining in up to 50% of cells or 3+ staining in up to 20% of cells were considered “weakly positive”. Tumors with 2+ staining in >50% or 3+ staining in >20% were considered “strongly positive”. The quantification of Ki67 was performed as previously described [17]. We scored the cancer focus with the highest labeling index. We set the cut-off for the Ki67 labeling index at 10% for positive tumor cells based on their Receiver Operating Characteristic.

**Figure 1:**
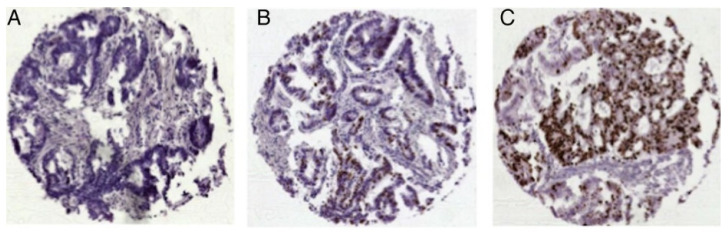
Representative images of Ki67 expression in colorectal cancer: (**A**) Ki67 low, (**B**) Ki67 moderate and (**C**) Ki67 high expression; magnification ×50 each. (Reprinted with permission from Ref. [7]. License: http://creativecommons.org/licenses/by-nc/4.0/, accessed on 1 June 2024).

A score was established using the results from the studies mentioned above [6,7,8]). Points were given to the various staining intensities according to their influence on survival in colon cancer. Strong H2BUB1 staining was allocated no points; one point was given for weak and two points for negative staining. RBM3 results were stratified into two groups with either positive (0 points) or negative (2 points) staining. A Ki67 labeling index > 10% received no points, while a Ki67 labeling index < 10% received one point. Thus, the score ranged from 0 to 5 (0 = low risk, 1–2 = intermediate risk, 3 = high risk, 4–5 = very high risk). Only those TMA spots that were analyzable for all three biomarkers were included in the analysis. Figure 1 shows Ki67 immunohistochemistry for colorectal cancer (Figure 1).

### 2.2. Statistics

Statistical calculations were performed with JMP^®^ 10.0.2 software (2012 SAS Institute Inc., Cary, NC, USA). Contingency tables and the chi-squared test were performed to search for associations between molecular parameters and tumor phenotype. Survival curves were calculated according to Kaplan–Meier. The Log-Rank test was applied to detect significant survival differences between groups. The Cox proportional hazards regression analysis was performed to test the statistical independence and significance between pathological, molecular and clinical variables. For this, examined parameters were pooled as the following:

3-biomarker classifier: high/very high risk vs. low/intermediate risk
pT-status: T3/4 vs. T1/2
Grade: G3/4 vs. G1/2
Nodal status: pN+ vs. pN0
Localization: right vs. left/transversum

## 3. Results

A significant association between clinicopathological data and the 3-biomarker classifier was found for tumor stage, grading and vascular invasion (*p* = <0.0001, *p* = 0.0065 and *p* = 0.0022, respectively). The lymph node metastases, histological type, localization of the tumor and peritumoral lymphocytes did not correlate with the score. Table 2 illustrates the association between the 3-biomarker classifier and histopathological data (Table 2).

### 3.1. Survival Analysis

In respect to the 3-biomarker classifier, the median overall survival (OS) in patients with high scores was significantly lower (scores 4–5: median OS 26 months; *p* = 0.0001) than in those with low scores.

**Figure 2:**
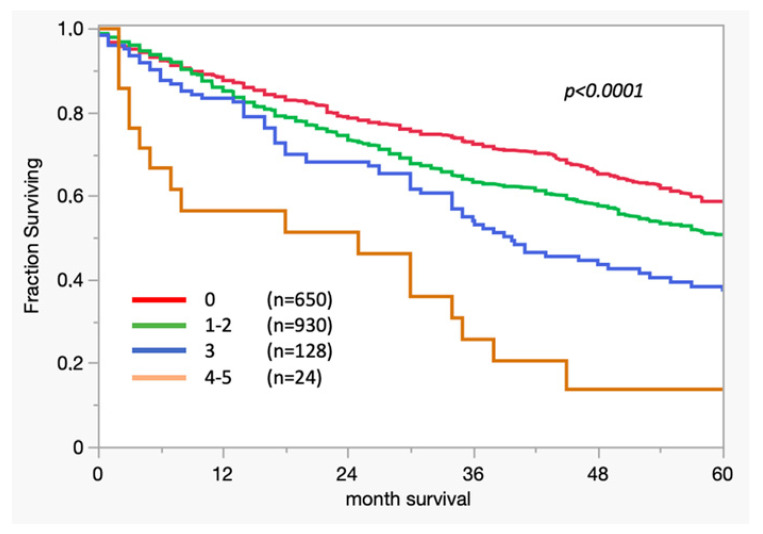
Kaplan–Meier curve: association between survival and 3-biomarker classifier results in colon cancer.

Figure 2 shows the Kaplan–Meier curves of the 3-biomarker classifier (Figure 2).

### 3.2. Cox Regression

Five variables were shown to be significant in respect to survival in the univariate analysis (tumor status, nodal status, 3-biomarker classifier, grading and tumor localization).

The multivariate analysis revealed that four out of these five variables, markedly the 3-biomarker classifier, were independent prognostic factors (tumor status, nodal status, 3-biomarker classifier and grading). Table 3 shows the results of the multivariable analysis (Table 3). When applying the 3-biomarker classifier, the risk of death is 1.45 times higher in the high-risk/very high-risk cohort than in the low/intermediate group (*p* = 0.0016).

## 4. Conclusions

In this study with a large patient cohort and corresponding clinicopathological data, we were able to show that the novel 3-biomarker classifier is able to predict outcomes in patients with CRC very simply. The patients with high scores had significantly worse survival than in the low-risk groups (*p* = 0.0001). Additionally, the hazard ratio for death is significantly higher in patients with high/very high-risk scores (HR 1.45). This score is obtainable using routine immunohistochemistry staining.

Currently, classical pathological TNM staging is used to perform a risk stratification of patients. Based on this, chemotherapy is recommended, e.g., for nodal positivity.

However, this classification system has its drawbacks. Approximately, 30% of patients with nodal negative pathological staging will develop metastases [5].

Currently, several biomarkers are being investigated to ensure a more individual approach. The integration of proteomic and genomic data has led to the identification of novel protein biomarkers for colorectal cancer. By analyzing the plasma proteome and integrating it with genomic data, key proteins involved in cancer progression have been identified, which can improve prognostic models and guide personalized treatment strategies [18].

Additionally, these advancements in identifying genomic biomarkers have significantly improved the prediction of treatment responses in colorectal cancer. A study identified several differentially expressed genes (DEGs) that are associated with the sensitivity to neoadjuvant chemoradiotherapy in locally advanced rectal cancer. These findings underscore the potential of integrating genomic biomarkers into clinical practice to enhance the precision of treatment planning and improve patient outcomes [19].

The early detection of colorectal cancer remains a critical challenge. Biomarkers developed through the Early Detection Research Network have shown promise in improving early detection rates. These biomarkers, particularly those identified through non-invasive tests, offer a practical approach to screening, which can lead to earlier diagnosis and better patient outcomes. The ongoing efforts to translate these biomarkers into clinical practice highlight their potential to reduce colorectal cancer mortality [20].

Furthermore, circular RNAs (circRNAs) have emerged as important biomarkers in colorectal cancer, with potential applications in diagnosis, prognosis and therapy. Their unique structure and regulatory functions make them stable biomarkers that can be detected in bodily fluids. Studies have demonstrated that specific circRNAs are associated with tumor progression and patient survival, suggesting their utility in non-invasive diagnostic tests and as targets for novel therapies [21].

One other way to overcome undertreatment due to nodal negativity might be in using techniques that increase the detection rate of occult lymph node metastases. Guanylyl cyclase C (GUCY2C), “an intestinal tumor suppressor” [22], has been described as a key factor of carcinogenesis in colorectal carcinoma. Furthermore, in nodal negative patients, the expression of GUCY2C might serve as a surrogate marker of occult metastases in lymph nodes [22]. Using GUCY2C as a marker, Mejia et al. were able to show that 87% of patients with pN0-staging expressed this receptor, suggesting occult lymph node metastases [23]. These data highlight the shortcomings of classical TNM staging and the need for other TNM-independent markers and scores. Stratification of patients according to their lymph node status, even if occult metastases were detected, also is not flawless since lymph node status is not the only predictive marker in colorectal carcinoma. Furthermore, there are controversies regarding tumor deposits (TD), which are classified as N1c in the current TNM staging system (8th edition). Wang et al. were able to show, “that the number of TDs was negatively correlated with the prognosis of CRC patients” [24]. This issue highlights the fundamental problem with the current pathological staging and makes other independent prognostic tools attractive.

Ahluwalia et al. were able to validate a genetic signature with four genes, which is able to predict prognosis in patients with colorectal carcinoma. Their multivariate analysis revealed that “a high prognostic score (composite 4 gene signature—DPP7/2, YWHAB, MCM4 and FBXO46) was found to be a significant predictor of poor prognosis in CRC patients (HR: 3.42, 95% CI: 1.71–7.94, *p* < 0.001 *)” [25]. The advantage of the molecular markers used in our cohort (RBM3, Ki-67 and H2BUB1) is their accessibility and abundant commercial availability, allowing for their routine laboratory investigation. The rationale behind combining the scores is to allow for better stratification between low- and high-risk colon cancer cohorts as compared to single parameters, such as nodal positivity or T-stage.

The validity of RBM3 as a prognostic marker was recently presented by Gao and colleagues. In a large meta-analysis, including 17 included studies comprising 4976 patients, they were able to show that the expression of RBM3 was significantly associated with improved overall, disease-free and recurrence-free survival in several malignancies. Furthermore, a subgroup analysis for patients with CRCs confirmed this finding with a HR of 0.61 [26]. On the other hand, Sugai et al. published their data regarding markers in submucosal colorectal cancer. In their study, the high expression of Ki-67 was significantly associated with lymph node metastases [27]. We must assume that the prognostic role of Ki-67 remains unclear and needs to be part of further studies. However, our previously published data regarding Ki-67 is based on a large database, which fortifies our hypothesis [7]. Concerning H2BUB1 and its role in CRC carcinogenesis, Tarcic et al. showed, in their experimental study, that “low H2BUB1 favors inflammation and cancer” [28]. This is congruent with our previously published data [8].

The 3-biomarker classifier presents a promising tool for prognostication in colorectal cancer, with potential implications for clinical decision-making. Implementing this method in clinical practice would require validation studies, the standardization of staining protocols, and integration into existing diagnostic workflows. This score could potentially guide treatment decisions, identifying high-risk patients who might benefit from more aggressive therapies. Compared to current treatment markers, the classifier could reclassify a significant number of patients, thereby influencing treatment strategies and improving patient outcomes.

As for this current study, some limitations must be considered. The retrospective design of this study makes it prone to recall and misclassification biases. Additionally, the current study lacks external validation and, therefore, might dampen the reliability of the results. Despite several predictive tools being available in the literature, their validation is lacking. In 2017, a systematic review from the Memorial Sloan Kettering was summarized as follows: “Prognostication tools are important devices for patient management, but tool reliability is compromised by poor quality” [29]. This highlights the importance of the external validation of the proposed markers.

Finally, checklists have been proposed for the improved reporting of prediction models (e.g., the Transparent Reporting of a multivariable prediction model for the Individual Prognosis or Diagnosis checklist) which were not used in this current study [30].

To ensure the stability and reproducibility of the 3-biomarker classifier, further studies involving repeated measurements and cross-laboratory validation are essential. Although not included in the current study, these steps are critical for clinical implementation. Future research should focus on processing high- and low-scoring samples across multiple labs to confirm score consistency.

On the other hand, the aforementioned limitations are clearly attenuated by the large cohort. Furthermore, the results from each single marker have been thoroughly evaluated on our large colorectal cancer tissue microarrays and the results were published in peer-reviewed journals.

Although H2BUB1, RBM3 and Ki-67 are not yet routinely performed in Europe, their commercial availability and established protocols for immunohistochemistry make them feasible for routine clinical implementation.

To conclude, the 3-biomarker classifier can serve as a potent tool to predict survival in patients with CRC. At this point in time, the score can already be implemented in clinical routines rather easily to classify patients with CRC. In the future and after external validation of the results, this score might facilitate shared decision making regarding adjuvant chemotherapy in nodal negative patients.

Our study demonstrates that the 3-biomarker classifier, incorporating H2BUB1, RBM3 and Ki-67, is an effective tool for predicting survival outcomes in patients with colorectal cancer. This classifier can identify high-risk patients who may benefit from more aggressive treatment, while sparing low-risk patients from unnecessary interventions. Implementing this risk score in clinical practice could improve personalized treatment strategies and overall patient outcomes.

## Figures and Tables

**Table 1:** Clinical and histopathological data of patients.

Clinical/Pathological Features		n Available
Gender	Female	898
	Male	893
Age	Mean: 69 (29–96)	
Tumor grade	G1	33
	G2	1497
	G3	242
Tumor stage	pT1	80
	pT2	283
	pT3	1143
	pT4	269
Nodal status	pN0	909
	pN1	448
	pN2	392
Tumor type	Tubular carcinoma	1264
	Mucinous carcinoma	119
	Others	22
Localization	Right colon	355
	Transverse colon	134
	Left colon	418
	Rectum	482
Total number of patients (n evaluable)		1791

**Table 2:** Association between 3-biomarker classifier results and colon cancer histopathological data.

3-Biomarker Classifier Risk						
Parameter	n Evaluable	Low (%)	Intermediate (%)	High (%)	Very High (%)	*p* Value
All cancers	2212	993 (44.9)	1061 (48.0)	130 (5.9)	28 (1.2)	
Tumor stage						<0.0001
						
pT1	80	47 (58.8)	31 (38.8)	2 (2.5)	0 (0.0)	
pT2	283	126 (44.5)	141 (49.8)	16 (5.7)	0 (0.0)	
pT3	1143	428 (37.4)	623 (54.5)	81 (7.1)	11 (1.0)	
pT4	269	74 (27.5)	154 (57.2)	28 (10.4)	13 (4.8)	
						
Lymph node metastasis						0.2559
						
pN0	909	353 (38.8)	492 (54.1)	58 (6.4)	6 (0.7)	
pN1	448	168 (37.5)	237 (52.9)	35 (7.8)	8 (1.8)	
pN2	392	142 (36.2)	208 (53.1)	32 (8.2)	10 (2.6)	
Grading						0.0065
						
G1	33	20 (60.6)	13 (39.4)	0 (0.0)	0 (0.0)	
G2	1497	550 (36.7)	821 (54.8)	109 (7.3)	16 (1.1)	
G3	242	105 (43.4)	114 (47.1)	17 (7.0)	6 (2.5)	
						
Tumor localization						0.2130
Right	355	132 (37.2)	184 (51.8)	29 (8.2)	10 (2.8)	
Transverse	134	42 (31.3)	72 (53.7)	16 (11.9)	4 (3.0)	
Left	418	148 (35.4)	240 (57.4)	25 (6.0)	5 (1.2)	
Rectum	482	173 (35.9)	267 (55.4)	37 (7.7)	5 (1.0)	
Histological type						0.1805
						
Adenocarcinoma	1264	442 (35.0)	704 (55.7)	96 (7.6)	21 (1.7)	
Mucinous	119	53 (44.5)	58 (48.7)	8 (6.7)	0 (0.0)	
Others	22	9 (40.9)	10 (45.5)	2 (9.1)	1 (4.5)	
Peritumoral lymphocytes						0.1453
						
Absent	787	297 (37.7)	427 (54.3)	52 (6.6)	11 (1.4)	
Present	602	198 (32.9)	339 (56.3)	54 (9.0)	11 (1.8)	
Vascular invasion						0.0022
						
No	783	299 (38.2)	428 (54.7)	50 (6.4)	6 (0.8)	
Yes	605	196 (32.4)	337 (55.7)	56 (9.3)	16 (2.6)	

**Table 3:** Cox proportional hazard regression analysis: hazard ratios accompanied by the corresponding *p*-values of five variables: 3-biomarker classifier, tumor status, grading, nodal status (N+ indicates presence of lymph node metastases) and tumor localization.

	3-Biomarker-Classifier	Tumor Status	Grading	Nodal Status	Tumor Localization
	High/Very High Risk vs. Low/Intermediate Risk	T3/4 vs. T1/2	G3/4 vs. G1/2	pN+ vs. pN0	Right vs. Left/Transversum
Hazard Ratio	1.45	2.23	1.32	2.49	1.00
*p*-value	0.0016	<0.001	0.0126	<0.001	0.9618

## Data Availability

The data presented in this study are available on request from the corresponding author.
